# Mitochondria-targeted antioxidant therapy with MitoQ ameliorates aortic stiffening in old mice

**DOI:** 10.1152/japplphysiol.00670.2017

**Published:** 2017-10-26

**Authors:** Rachel A. Gioscia-Ryan, Micah L. Battson, Lauren M. Cuevas, Jason S. Eng, Michael P. Murphy, Douglas R. Seals

**Affiliations:** ^1^Department of Integrative Physiology, University of Colorado, Boulder, Boulder, Colorado; ^2^MRC Mitochondrial Biology Unit, University of Cambridge, Cambridge, United Kingdom

**Keywords:** aging, artery, mitochondrial antioxidant

## Abstract

Aortic stiffening is a major independent risk factor for cardiovascular diseases, cognitive dysfunction, and other chronic disorders of aging. Mitochondria-derived reactive oxygen species are a key source of arterial oxidative stress, which may contribute to arterial stiffening by promoting adverse structural changes—including collagen overabundance and elastin degradation—and enhancing inflammation, but the potential for mitochondria-targeted therapeutic strategies to ameliorate aortic stiffening with primary aging is unknown. We assessed aortic stiffness [pulse-wave velocity (aPWV)], ex vivo aortic intrinsic mechanical properties [elastic modulus (EM) of collagen and elastin regions], and aortic protein expression in young (~6 mo) and old (~27 mo) male C57BL/6 mice consuming normal drinking water (YC and OC) or water containing mitochondria-targeted antioxidant MitoQ (250 µM; YMQ and OMQ) for 4 wk. Both baseline and postintervention aPWV values were higher in OC vs. YC (post: 482 ± 21 vs. 420 ± 5 cm/s, *P* < 0.05). MitoQ had no effect in young mice but decreased aPWV in old mice (OMQ, 426 ± 20, *P* < 0.05 vs. OC). MitoQ did not affect age-associated increases in aortic collagen-region EM, collagen expression, or proinflammatory cytokine expression, but partially attenuated age-associated decreases in elastin region EM and elastin expression. Our results demonstrate that MitoQ reverses in vivo aortic stiffness in old mice and suggest that mitochondria-targeted antioxidants may represent a novel, promising therapeutic strategy for decreasing aortic stiffness with primary aging and, possibly, age-related clinical disorders in humans. The destiffening effects of MitoQ treatment may be at least partially mediated by attenuation/reversal of age-related aortic elastin degradation.

**NEW & NOTEWORTHY** We show that 4 wk of treatment with the mitochondria-specific antioxidant MitoQ in mice completely reverses the age-associated elevation in aortic stiffness, assessed as aortic pulse-wave velocity. The destiffening effects of MitoQ treatment may be at least partially mediated by attenuation of age-related aortic elastin degradation. Our results suggest that mitochondria-targeted therapeutic strategies may hold promise for decreasing arterial stiffening with aging in humans, possibly decreasing the risk of many chronic age-related clinical disorders.

## INTRODUCTION

Advancing age is a primary risk factor for the development of numerous chronic degenerative diseases, which are the leading causes of morbidity and mortality in the United States and other developed nations (20, 30, 41). A key event underlying the etiology of many chronic age-related disorders is stiffening of the large elastic arteries, specifically, the aorta. Elevated aortic stiffness increases the pulsatile shear and pressure experienced by the heart, blood vessels, and other organs, which can have numerous pathophysiological effects contributing to the development of disease (23, 32, 34, 35, 38, 62). Indeed, aortic pulse-wave velocity (aPWV), the gold-standard measure of arterial stiffness, is a strong independent risk factor for incident cardiovascular events among older adults (34, 50), and it also predicts the development of chronic kidney disease, stroke, cognitive impairment, and Alzheimer disease (2, 7, 18, 21, 43, 53). Current demographic trends forecast a major increase in the number of older adults in the coming decades, which will be accompanied by attendant increases in disease prevalence and health care costs (19, 22, 56). As such, a top biomedical research priority is to identify strategies that prevent or reverse aortic stiffening with advancing age, as this may help prevent, reduce, or delay the development of multiple common disorders of aging.

A key mechanism underlying the development of age-related arterial stiffening may be vascular mitochondrial oxidative stress and associated excessive production of mitochondria-derived reactive oxygen species (mtROS). Mitochondria are now recognized as a primary source of arterial oxidative stress with aging and cardiovascular diseases (1, 4, 5, 16, 31, 38, 55, 61), and evidence from genetic models indicates that experimental modulation of mtROS affects large elastic artery stiffening. For example, age-related arterial stiffening, pathological remodeling, and vascular disease are accelerated in mice deficient in the mitochondrial antioxidant protein manganese superoxide dismutase (SOD2) (61). In support of a role specifically for mitochondria-derived oxidative stress, selective deletion of a cytosolic isoform of prooxidant enzyme NADPH oxidase (NOX1/2)—with the mitochondrial isoform (NOX4) intact—does not prevent age-related arterial stiffening in the setting of atherosclerosis (55), implicating mtROS as a key driver of age-related arterial pathology.

Excessive levels of arterial mtROS may promote arterial stiffness via redox-related alterations in structural protein turnover and through induction of proinflammatory signaling. Changes in arterial wall structure are a major mechanism by which the large elastic arteries stiffen with age (9, 17, 24, 32, 62); specific structural alterations include increased deposition of the load-bearing protein collagen and degradation and fragmentation of elastin (17, 24, 42). Oxidative stress, including that derived specifically from mitochondria, alters the activity of the enzymes involved in structural protein turnover and shifts the balance of synthesis and breakdown toward collagen deposition and elastin degradation (9, 17, 24, 38, 55, 61, 62), contributing to dysregulation of structural protein homeostasis and consequent arterial stiffening.

Mitochondria-derived ROS are also emerging as important for promoting and sustaining arterial inflammation, a hallmark of arterial aging and critical mediator of arterial stiffening (24, 38, 39, 57). A proinflammatory environment in the vasculature, secondary to excessive mtROS production, may contribute to arterial stiffening through many mechanisms, including induction of gene expression patterns that alter structural protein turnover, impairment of vascular endothelial function, increases in vascular smooth muscle cell tone, and further invasion of the vascular wall by proinflammatory mediators that also reinforce oxidative stress (24, 31, 32, 39, 57, 61).

Our laboratory recently demonstrated that treating old mice with the mitochondria-targeted antioxidant MitoQ to lower mitochondrial oxidative stress completely reversed the age-related impairment in arterial endothelial function in old mice (15). However, the effects of mitochondria-targeted antioxidants on aortic stiffness with primary aging have never been investigated. Therefore, in this study, we tested the hypothesis that 4 wk of MitoQ supplementation in the drinking water would decrease aortic stiffness (as assessed in vivo by aPWV) in old mice. To gain insight into the potential underlying mechanisms, we also assessed the collagen- and elastin-mediated contributions to intrinsic aortic stiffness (assessed ex vivo in aortic rings), aortic protein expression of these key structural proteins, and aortic expression of inflammatory cytokines.

## METHODS

All studies were approved by the Institutional Animal Care and Use Committee at the University of Colorado Boulder and conformed to the *Guide for the Care and Use of Laboratory Animals* (National Research Council, 2011).

### 

#### Mice.

Male C57BL/6 mice, an established model of age-related vascular dysfunction (15, 48), were purchased from the aging colony at the National Institute on Aging at ~4 or ~25 mo of age and allowed to acclimate to our facilities for 2 wk before beginning treatment. Mice were housed in standard cages on a 12:12-h light-dark cycle and were allowed access to normal rodent chow (Harlan 7917) and water ad libitum. Body mass and water intake were monitored regularly throughout the study.

#### MitoQ treatment.

On the basis of reports of effective dose and duration of treatment with MitoQ and our previous work (15, 40, 46), mice were randomly assigned to treatment with MitoQ [250 µM; in the form of Mitoquinone mesylate adsorbed to β-cyclodextrin (~22% MitoQ by weight) from Antipodean Pharmaceuticals, San Francisco, CA]. Young MitoQ-treated (YMQ, ~6 mo., *n* = 11) and old MitoQ-treated (OMQ, ~27 mo, *n* = 10) mice were compared with mice provided normal drinking water [young control (YC, ~8 mo, *n* = 8) and old control (OC, ~27 mo, *n* = 10)] for 4 wk, a duration we have previously shown to be effective in reversing age-related arterial endothelial dysfunction (15). MitoQ was prepared fresh (the preparation is water-soluble) and administered in light-protected water bottles changed every 3 days.

#### In vivo assessment of arterial stiffness: aortic pulse-wave velocity.

In vivo arterial stiffness was assessed at baseline and after4 wk of MitoQ treatment by aortic pulse-wave velocity (aPWV) using Doppler ultrasound, as previously described by our laboratory (11, 28). Briefly, mice were anesthetized via inhaled isoflurane (1.5–2%) and positioned supine on a warmed platform with paws secured to electrocardiogram leads. Doppler probes were placed at the transverse aortic arch and abdominal aorta to detect pulse waves. Three consecutive 2-s recordings were made for each animal and used to determine time delay between the electrocardiogram R-wave and the foot of the Doppler signal for each site (Δtime_abdominal_ and Δtime_transverse_). aPWV was then calculated as aPWV = (physical distance between the two probes)/(Δtime_abdominal_ − Δtime_transverse_) and reported in centimeters per second.

To examine the potential role of changes in blood pressure to treatment-related differences in aPWV, we assessed systolic and diastolic blood pressure at baseline and after 4 wk of MitoQ or normal drinking water consumption using the CODA noninvasive tail-cuff system, as previously described (11, 28). The pressure measurements from 20 collection cycles (following five acclimation cycles) on each of three consecutive days were averaged for each mouse at each time point.

#### Ex vivo assessment of arterial stiffness: intrinsic mechanical stiffness.

After all in vivo assessments were completed, mice were euthanized and aortas were harvested for measurements of ex vivo intrinsic mechanical stiffness and protein expression. Two 1-mm aortic rings from the thoracic region (dissected free of surrounding connective tissue) were used to assess intrinsic aortic stiffness via wire myography, as described previously by our laboratory (6, 10, 14, 28). Aortic rings were loaded into heated myograph chambers (Danish Myo Technology, Aarhus, Denmark) with calcium-free PBS. After three cycles of prestretching were completed, ring diameter was increased to achieve 1 mN force and then incrementally stretched by ~10% every 3 min until failure. The force corresponding to each stretching interval was recorded and used to calculate stress and strain, defined as follows: Strain (λ) = Δ *d*/*d*(*i*), where *d* is diameter, *d*(*i*)  is  initial diameter; Stress (*t*) = λ*L*/2*HD*, where *L* is one-dimensional load, *H* is wall thickness determined by histology, and *D* is vessel length.

The slope of the stress-strain curve was used to determine the elastic modulus in the collagen-dominant and elastin-dominant regions of the curve, as described below.

#### Collagen elastic modulus.

When aortic rings are subjected to stress-strain testing, the region of the stress-strain curve corresponding to the highest forces represents the stretching of predominantly collagen fibers (25, 47). The elastic modulus of the collagen-dominant region was determined as the slope of the linear regression fit to the final four points of the stress-strain curve, as described previously (6, 14, 28). See (Fig. 2) for representative stress-strain curve.

#### Elastin elastic modulus.

During stress-strain testing in aortic rings, the region of the stress-strain curve corresponding to the stretching of exclusively elastin fibers is a lower-force region before collagen fiber engagement that can be identified as the portion of the stress-strain curve where curvature (determined from the second derivative of the stress-strain curve) is approximately zero; the engagement of collagen fibers is indicated by an elevation in the curvature (nonzero second derivative) (25). To determine the boundaries of the elastin region of our stress-strain curves, we calculated the roots of the second derivative of a 7th-order polynomial fit to the data (*R*^2^ > 0.99). The first root was considered the boundary between the very low-force region and the elastin region, and the second root was considered the boundary between the elastin region and the onset of collagen fiber engagement (25). The elastic modulus of the elastin region was then determined as the slope of the linear regression fit to the stress-strain data between the two points. See Fig. 2 for the representative stress-strain curve.

#### Aortic protein expression.

Aortic expression of structural proteins collagen-I and α-elastin was determined in aortic homogenates by standard Western blot techniques and immunohistochemistry (IHC) in aortic sections, as previously described (6, 11, 28). Aortic protein expression of inflammatory cytokines was determined using a custom multiplex ELISA (Ciraplex, Aushon Biosystems, Billerica, MA), as previously described (27, 29).

Before Western blot analysis and cytokine multiplex, aortas were homogenized in radio-immunoprecipitation assay lysis buffer, and protein concentration was determined using the Pierce BCA assay kit (ThermoFisher Scientific, Fremont, CA).

For Western blot analysis, 15 µg of aortic protein were loaded onto 4–12% polyacrylamide gels and then transferred onto nitrocellulose membranes (Criterion System; Bio-Rad, Hercules, CA). Membranes were incubated (overnight at 4°C) with primary antibodies: collagen-I (1:1,000; Millipore, Burlington, MA), α-elastin (1:200; Abcam, Cambridge, MA), and glyceraldehyde 3-phosphate dehydrogenase (1:1,000, normalizer, GAPDH; Cell Signaling, Danvers, MA). Proteins were visualized on a digital acquisition system (ChemiDoc-It, UVP, Upland, CA) using chemilluminescence with horseradish peroxidase-conjugated secondary antibodies (Jackson ImmunoResearch, West Grove, PA) and ECL substrate (Pierce, Rockford, IL). Relative intensity was quantified using ImageJ software and normalized to GAPDH intensity (obtained from the same blots after stripping) and then expressed as a ratio of the mean intensity of the young control group.

For cytokine multiplex, 15 μg of aortic lysate were loaded into microplate wells, and an assay was performed according to the manufacturer's instructions. The multiplex plates were custom designed (custom Ciraplex, Aushon) for detection of the following murine proinflammatory cytokines: IL-1β, IL-6, IL-10, and IFN-γ. Images were captured using Cirascan imager (Aushon), and results were analyzed with Cirasoft software (Aushon). If levels of a given cytokine were undetectable (e.g., fell below the limit of detection of the assay), samples were excluded from the analysis.

For IHC, ~1-mm thoracic aortic segments were frozen in OCT compound in liquid nitrogen-cooled isopentane before sectioning. Aortic sections (7 µm) were fixed in acetone, washed in Tris buffer, and stained using the Dako EnVision+ System-HRP-DAB kit, as performed previously in our laboratory (11). Sections were incubated for 1 h at 4°C with primary antibodies for α-elastin (1:50; Abcam) or collagen-I (1:200; Millipore) and then incubated with the labeled polymer secondary for 30 min. Slides were dehydrated and coverslipped after a 10-min or 1-min exposure to diaminobenzidine (elastin and collagen, respectively).

Stained aortic sections were imaged using a Nikon Eclipse TS100 photomicroscope under identical conditions. Quantification of the integrated density of the stain was performed using ImageJ software by a single investigator blinded to the group assignment of each sample. Collagen-I expression was assessed in the whole artery sections, comprising both the medial and adventitial layers, whereas elastin expression was assessed in the medial layer, the primary site of age-related changes in elastin expression (9, 10). Integrated density values from four sections were averaged to provide a single value for each protein per aorta, which are expressed relative to the mean of the young control group.

#### Statistical analysis.

All statistical analyses were performed using SPSS 23.0 software (Armonk, NY). Data were first assessed for outliers and normality/homogeneity of variance. Between-group differences in morphological characteristics and aortic protein expression (Western blot, immunohistochemistry, and multiplex ELISA) were determined using one-way ANOVA. Between-group differences in elastic modulus (collagen and elastin regions) were determined using a linear mixed model with age (young vs. old) and treatment (control vs. MitoQ) as factors, whereas within-group differences in aPWV and blood pressure were examined using a linear mixed model that also included a repeated factor (preintervention vs. postintervention period). When a significant main effect was observed, Fisher’s least significant difference post hoc tests were performed to determine specific pair-wise differences.

## RESULTS

MitoQ consumption across the 4-wk treatment period was similar to our previous report and not different between young and old mice (~1 mmol/day; Ref. 15). Select morphological characteristics and blood pressure are shown in Table 1. Consistent with our previous study (15), 4 wk of MitoQ treatment did not influence overall morphology; although there were age-associated differences in body mass, heart mass, and quadriceps mass, these were not different between mice receiving MitoQ vs. normal drinking water. There were no age- or treatment-related differences in aortic diameter or systolic and diastolic blood pressure.

**Table 1.** General morphological characteristics and blood pressure

	YC	OC	YMQ	OMQ
Body mass, g	25.1 ± 1.2	29.4 ± 2.7*	26.0 ± 1.4	28.5 ± 3.0*
Heart mass, mg	128 ± 11	175 ± 22*	124 ± 9	164 ± 20*
Liver mass, g	1.34 ± 0.06	1.41 ± 0.16	1.34 ± 0.18	1.37 ± 0.38
Quadriceps mass, mg	163 ± 28	138 ± 27*	175 ± 29	143 ± 27*
Visceral fat mass, mg	306 ± 70	302 ± 89	256 ± 71	229 ± 118
Aorta diameter, µm	749 ± 72	780 ± 47	789 ± 77	785 ± 43
Systolic BP, mmHg	Pre: 105.1 ± 10.1	Pre: 101.3 ± 11.7	Pre: 101.2 ± 6.7	Pre: 93.5 ± 10.8
	Post: 101.4 ± 12.9	Post: 94.9 ± 5.0	Post: 98.2 ± 10.8	Post: 101.0 ± 4.4
Diastolic BP, mmHg	Pre: 73.3 ± 11.5	Pre: 72.8 ± 11.0	Pre: 73.1 ± 4.7	Pre: 66.3 ± 4.2
	Post: 71.9 ± 11.3	Post: 67.0 ± 4.6	Post: 74.1 ± 9.3	Post: 71.9 ± 10.1

Data are presented as means ± SD. YC, young control mice; OC, old control mice; YMQ, young MitoQ-treated mice; OMQ, old MitoQ-treated mice; BP, blood pressure; Pre, baseline assessment (before treatment period); Post, assessment following 4-wk treatment period with MitoQ or normal drinking water.

* *P* < 0.05 vs. YC and YMQ.

### 

#### MitoQ treatment reverses aortic stiffening in old mice.

**Fig. 1.**
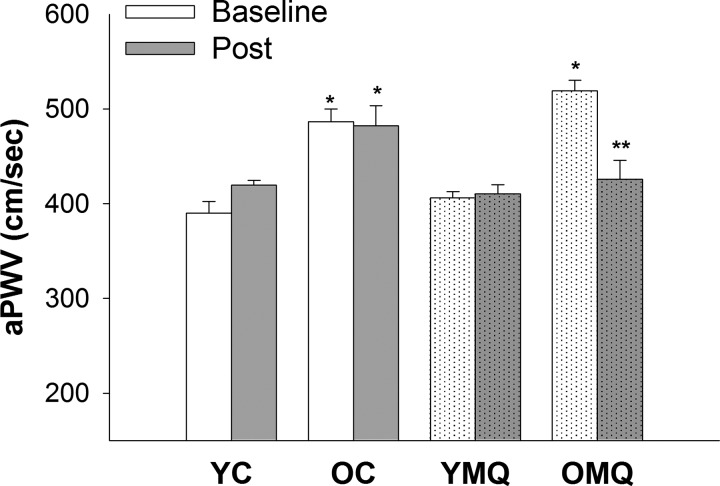
MitoQ treatment reverses age-related aortic stiffness in mice. Aortic pulse-wave velocity (aPWV) was assessed in young and old mice before (baseline) and after (post) consumption of normal drinking water (YC and OC) or MitoQ treatment (YMQ and OMQ) for 4 wk. *n* = 8–11/group; error bars represent means ± SE *P < 0.05 vs. YC and YMQ; **P < 0.05 vs. OC and OMQ baseline.

At baseline, aPWV was significantly higher in old compared with young mice, and aPWV was not significantly different from baseline to postintervention in either young or old control mice receiving normal drinking water (Fig. 1). In contrast, 4 wk of MitoQ treatment significantly decreased aPWV in old mice to levels similar to young mice following the intervention period. MitoQ treatment had no effect on aPWV in young mice. These results indicate that 4 wk of MitoQ treatment specifically reverses aortic stiffening in old mice.

#### Potential mechanisms underlying the destiffening effects of MitoQ treatment in old mice.

In our previous study using MitoQ treatment in old mice (15), the same dose and duration of treatment as used in the present study normalized the age-related elevation in aortic whole cell and mitochondria-specific superoxide production, indicating a profound antioxidant effect of MitoQ in arteries. To investigate further how decreased levels of mtROS in aging arteries may contribute to the destiffening effects of MitoQ, in the present study, we investigated key mechanisms that have been implicated downstream of mitochondrial oxidative stress in the development of age-related arterial stiffening, namely, changes in arterial structural proteins and inflammation.

#### Ex vivo aortic stiffness—collagen- and elastin-mediated mechanical properties of aortic rings.

The elastic modulus of the collagen region of stress-strain curves was significantly greater in old control vs. young control mice (Fig. 3*A*), whereas the elastic modulus of the elastin region was significantly lower in old control compared with young control mice (Fig. 3*B*), indicating an age-related increase in intrinsic arterial stiffness mediated by increased collagen and reduced elastin. MitoQ treatment had no effect on the collagen elastic modulus, such that the values in old and young MitoQ-treated mice were not significantly different from old and young control mice, respectively. However, in arteries from old mice treated with MitoQ, the elastic modulus in the elastin region was significantly greater than that of old control mice but remained significantly lower than the elastin elastic modulus of young MitoQ-treated mice, indicating attenuation of the age-related decline in elastin.

**Fig. 2.**
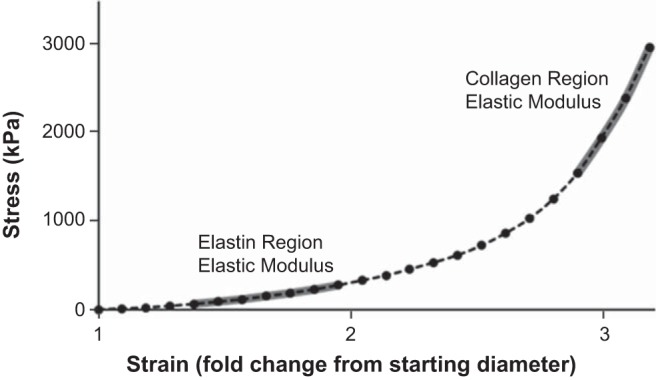
Representative stress-strain curve for determination of ex vivo intrinsic mechanical stiffness of aortic rings. Aortic rings were incrementally stretched until tissue failure, as described in materials and methods, and the tension (stress, kPa) corresponding to each stretch was plotted against strain (change in length relative to resting length) to generate a stress-strain curve. The elastic modulus of the region of the curve corresponding to collagen fiber stretching was determined as the slope of the line fit to the final four points on the curve before tissue failure (collagen region elastic modulus). The region of the curve corresponding to elastin fiber stretching was considered to lie between the very low-force region and the onset of collagen fiber engagement, which were identified as the first and second roots, respectively, of a seventh-order polynomial fit to the stress-strain curve (25). The elastic modulus of the elastin region of the curve was determined as the slope of the line fit between these boundaries (elastin region elastic modulus).

**Fig. 3.**
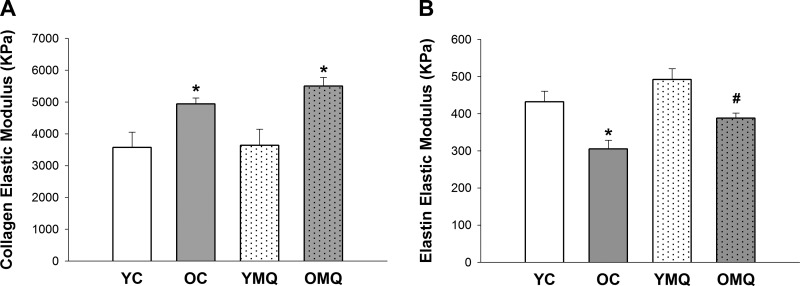
MitoQ treatment attenuates the age-related decline in elastin-mediated intrinsic mechanical properties but has no effect on collagen-mediated intrinsic mechanical stiffness. *A*: collagen region elastic modulus of aortic segments from young and old control (YC and OC) and young and old MitoQ-treated (YMQ and OMQ) mice. *B*: elastin region elastic modulus of aortic segments from YC, OC, YMQ, and OMQ mice. *n* = 8–11/group; error bars represent means ± SE. *P < 0.05 vs. YC and YMQ. #*P* < 0.05 vs. OC and YMQ.

#### Aortic expression of structural proteins.

**Fig. 4.**
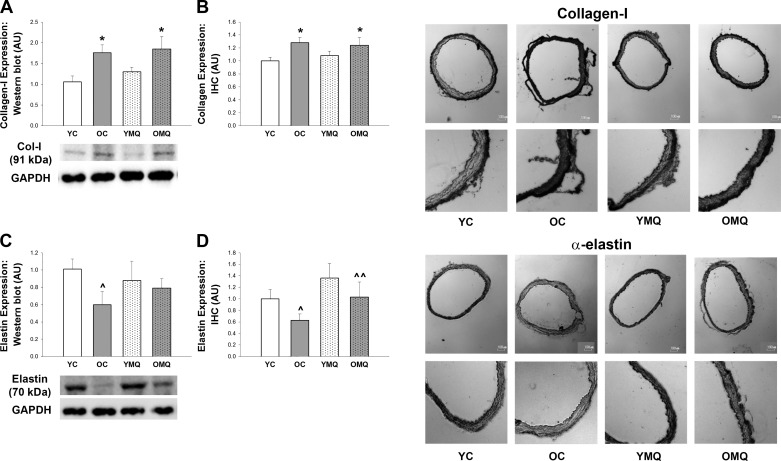
MitoQ treatment attenuates the age-related reduction in aortic elastin expression but has no effect on aortic collagen expression. *A*: aortic collagen-I expression assessed by Western blot analysis in aortic homogenates from young and old control (YC and OC) and young and old MitoQ-treated (YMQ and OMQ) mice. Expression levels are presented normalized to GAPDH expression and relative to the mean of the YC group (error bars represent means ± SE). Representative images, comprising four continuous lanes, are presented below mean data. The collagen-I and GAPDH images represent the same segment of the same blot. Any adjustments to the images were limited to changes in brightness and contrast made using ImageJ to optimize visualization, performed uniformly on the entire image. *n* = 6/group. *P < 0.05 vs. YC. *B*: aortic collagen-I expression assessed by immunohistochemistry in whole aortic sections from YC, OC, YMQ, and OMQ mice. Expression levels are presented relative to the mean of the YC group (error bars represent means ± SE). Representative images (whole sections and enlargements of the same sections) are presented to the right of the mean data. *n* = 7–11/group. *P < 0.05 vs. YC. *C*: aortic elastin expression assessed by Western blot analysis in aortic homogenates from young and old control (YC and OC) and young and old MitoQ-treated (YMQ and OMQ) mice. Expression levels are presented normalized to GAPDH expression and relative to the mean of the YC group (error bars represent means ± SE). Representative images, comprising four continuous lanes, are presented below mean data. The elastin and GAPDH images represent the same segment of the same blot. Any adjustments to the images were limited to changes in brightness and contrast made using ImageJ to optimize visualization, performed uniformly on the entire image. *n* = 6/group. ^*P* < 0.074 vs. YC. *D*: aortic elastin expression assessed by immunohistochemistry in the medial layer of aortic sections from YC, OC, YMQ, and OMQ mice. Expression levels are presented relative to the mean of the YC group (error bars represent means ± SE). Representative images (whole sections and enlargements of the same sections) are presented to the right of the mean data. *n* = 8–11/group. ^*P* = 0.086 vs. YC; **^^***P* = 0.075 vs. OC.

Consistent with our intrinsic mechanical stiffness observations, aortic collagen protein expression was significantly greater (Fig. 4, *A* and *B*), and aortic elastin expression was lower (Fig. 4, *C* and *D*, *P* = 0.074 and 0.086, respectively) in old control versus young control mice. MitoQ treatment did not affect aortic collagen content, such that collagen expression in old MitoQ-treated mice was not significantly different than that of old control mice, whether assessed in whole artery homogenate by Western blot analysis or in aortic sections via IHC. When measured in whole artery homogenate by Western blot analysis, aortic elastin levels in old MitoQ-treated mice were intermediate between (and not significantly different from) those of either young control or old control mice. However, when assessed via IHC in the medial layer of aortas—the primary site of age-related elastin degradation (9, 10)—elastin content in old MitoQ-treated mice was greater than that of old control mice (*P* = 0.07).

Together with our observations of intrinsic mechanical properties, these results suggest that the reduction in in vivo aortic stiffening in old mice after MitoQ treatment was mediated not by effects on aortic collagen, but possibly by partial preservation of elastin.

#### Aortic inflammatory cytokine expression.

**Fig. 5.**
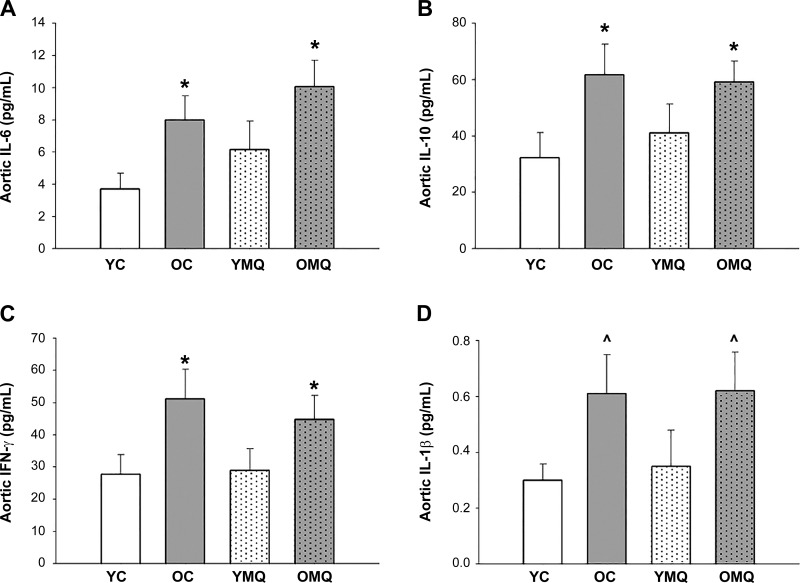
MitoQ treatment does not affect the age-related increase in aortic inflammatory cytokines. Expression of inflammatory cytokines IL-6 (*n* = 7–10/group) (*A*), IL-10 (*n* = 7–9/group) (*B*), IFN-γ (*n* = 7–10/group) (*C*), and IL-1β (*n* = 4–10/group) (*D*) in aortic homogenates from young and old control (YC and OC) and young and old MitoQ-treated (YMQ and OMQ) mice. Sample sizes reflect all aortic homogenates for which cytokine levels were detectable; samples were excluded when cytokine levels were undetectable/below the limit of quantification of the assay. Error bars represent means ± SE. *P < 0.05 vs. YC. ^0.10 > *P* > 0.05 vs. YC (*P* = 0.08, OC vs. YC; *P* = 0.06, OMQ vs. YC).

Aortic expression of proinflammatory cytokines IL-6, IL-10, and IFN-γ (Fig. 5, *A–C*) was significantly higher, and expression of IL-1β (Fig. 5*D*) tended to be higher, in old compared with young control mice, consistent with previous investigations demonstrating elevated levels of arterial cytokines with aging and association with vascular dysfunction (3, 27, 29, 44). Cytokine levels were not affected by 4 wk of MitoQ treatment (*P* > 0.05 OMQ vs. OC for all cytokines), suggesting that the destiffening effects of MitoQ were not mediated by changes in these aortic cytokines. However, these results do not preclude the possibility that MitoQ treatment may have influenced other components of inflammatory signaling pathways.

## DISCUSSION

The primary, novel finding of this study is that 4 wk of treatment with the mitochondria-targeted antioxidant MitoQ in old mice completely reverses the age-associated increase in aortic stiffness, assessed in vivo as aPWV. Our observation that MitoQ treatment decreases aortic stiffness in old mice extends previous work with general antioxidant compounds and adds to the evidence from transgenic and disease models that specifically implicate mitochondrial oxidative stress as a key contributor to aortic stiffening. A previous preclinical intervention study from our laboratory using the general antioxidant compound TEMPOL established oxidative stress as a key mechanism underlying age-related aortic stiffening (12), and other strategies that decrease arterial oxidative stress also ameliorate arterial stiffness (11, 13, 14, 28, 49). Recent work with genetic and disease models indicates that mitochondria are a major source of the vascular oxidative stress contributing to arterial stiffness. Mice with genetic deletion of mitochondrial antioxidant enzyme SOD2, a model of excess mitochondrial oxidative stress, demonstrate exacerbation of age-related aortic stiffening (61), and progression of age-related arterial stiffening is unaffected in mice with genetic deletion of cytosolic prooxidant NADPH oxidase (NOX1/2) but intact mitochondria-localized NADPH oxidase (NOX4) (55). Our finding here that in vivo treatment with the mitochondria-targeted antioxidant MitoQ in old mice decreases aortic stiffness provides further support for mitochondrial oxidative stress as a key mediator of arterial dysfunction with primary aging. Most importantly, our results extend previous observations from genetic and disease models (55) by demonstrating that a pharmacological intervention targeting excessive mtROS production reverses aortic stiffening in the setting of primary aging in mice, thus establishing an essential platform for translation to humans.

To gain initial mechanistic insight into the destiffening effects of MitoQ treatment, we assessed intrinsic mechanical stiffness ex vivo in aortic rings and examined both the collagen- and elastin-predominant regions of the stress-strain curves. In contrast to previous studies showing that the destiffening effects of late-life interventions, including those associated with decreased whole cell and mitochondrial oxidative stress, are primarily mediated by decreases in arterial collagen content (9, 11, 12, 14, 37, 55), we observed that MitoQ treatment had no significant effect on the collagen region elastic modulus or aortic collagen expression but instead attenuated the age-related decline in aortic elastin region elastic modulus and tended to preserve elastin expression. Our finding of partial elastin preservation with MitoQ treatment is consistent with the observations that heterozygous SOD2-deficient mice, a model of excess mtROS, show marked exacerbation of age-associated declines in arterial elastin content (61) and that lifelong caloric restriction, a setting of lower mtROS (26), preserves arterial elastin content with aging (8). Collectively, our results suggest that decreasing mitochondrial oxidative stress may at least partially preserve elastin content in the aorta, contributing to lower levels of stiffness.

Future studies are warranted to elucidate the mechanisms by which decreased mitochondrial oxidative stress (via MitoQ treatment) may preserve aortic elastin content in aging. One possible link may be mtROS-mediated regulation of enzymes that govern elastin turnover, including matrix metalloproteinases (MMP; Refs. 36 and 62)—changes in the activity of which are associated with arterial stiffening in both mouse models and human aging (32, 33, 58). For example, increased levels of MMP-2, a key enzyme involved in elastin degradation (9, 17, 59), accompany the loss of arterial elastin in heterozygous SOD2 knockout mice (61). Further, primary aging in preclinical models is associated with increased arterial MMP-2 expression (9, 59), and elevated aortic MMP-2 levels are also observed in human aging (33). Collectively, these previous studies suggest that age-related increases in mtROS may contribute to arterial elastin degradation via increased MMP-2 activity and that targeting excess mtROS, e.g., via MitoQ treatment, may attenuate elastin degradation, preserving elastin content in large elastic arteries and contributing to lower levels of stiffness. Although our results do not support a role for MitoQ in decreasing total arterial collagen content, future studies could examine not only arterial content of this key structural protein, but also changes in collagen fiber orientation (17) and formation of cross-links among proteins, both of which have the potential to influence arterial stiffness (9, 23, 62).

It is also important to consider mechanisms other than preservation of aortic elastin content that may have contributed to the dramatic decrease in aortic stiffness we observed with MitoQ treatment in old mice. In addition to structural changes, age-related arterial stiffening is also mediated by hemodynamic factors (including age-related reductions in vascular endothelial function) and increased vasomotor tone (17, 24, 62). Although our data indicate that changes in resting blood pressure did not contribute to the effects of MitoQ treatment, it is plausible that some of the destiffening that we observed in old mice was due to improvements in vascular endothelial function. Our previous study (15) demonstrated that MitoQ treatment increases endothelium-dependent dilation and nitric oxide bioavailability in old mice, both of which are important direct (e.g., effects on pulse pressure and smooth muscle tone) and indirect (e.g., nitric oxide, regulation of structural protein turnover) mediators of large elastic artery stiffness in vivo (17, 32, 38, 60, 62).

Aortic inflammatory cytokine levels were significantly elevated in aortic tissue of old vs. young mice, consistent with previous studies (27, 29, 44). Chronic low-grade arterial inflammation with aging, primarily mediated by NF-κB activation, can be triggered by excessive oxidative stress—including that derived from mitochondria—in a reciprocally reinforcing process that serves to impair arterial function (3, 29, 54). Although there is some evidence for a role of mtROS in mediating arterial inflammation and consequent dysfunction in atherosclerosis/disease models (31, 55), our observations in the present study do not support an anti-inflammatory role for MitoQ in reversing arterial stiffening in primary aging. After 4 wk of MitoQ treatment, there was no difference between old control and old MitoQ-treated aortic cytokine levels, despite the pronounced reversal of arterial stiffening in the latter. This suggests that the destiffening effects of MitoQ were mediated by a mechanism other than normalization of the aortic cytokines that we assessed here. However, it remains possible that MitoQ treatment influenced other components of inflammatory signaling, and future studies are warranted to investigate these possibilities.

Although the present study investigated the therapeutic efficacy of MitoQ in the setting of existing age-related aortic stiffness, it would also be of clinical relevance to determine whether targeting/decreasing mtROS earlier in life before the onset of aortic stiffening could prevent or slow the progression of pathological aortic remodeling and consequent cardiovascular sequelae. Given that excess mtROS are implicated as a key factor in the pathogenesis of numerous age-related conditions, including vascular dysfunction (1, 4, 5), it is possible that limiting an age-related increase in mtROS via treatment in early or midlife could prevent aortic stiffening. This possibility is supported by work from disease and senescence models, indicating that mitochondria-targeted therapeutics initiated before or at the onset of experimental insult or injury can prevent development or slow progression of dysfunction (45, 51, 52). Because low, physiological levels of mtROS are critical for the maintenance of cellular homeostasis, any optimal long-term therapeutic strategy would likely need to maintain mtROS at physiological levels rather than eliminate them completely.

### 

#### Conclusion.

In conclusion, the present study demonstrates that late-life treatment with a mitochondria-targeted antioxidant, MitoQ, effectively reverses aortic stiffening in the setting of primary aging. Our results suggest that this effect is mediated at least partially by attenuation/reversal of the age-related reduction in aortic elastin content, but additional work is needed to conclusively determine the mechanism(s) underlying the destiffening effect of MitoQ. Importantly, these results indicate that mitochondria-targeted antioxidants may represent a novel, promising therapeutic strategy for decreasing aortic stiffness, and potentially decreasing the risk of multiple chronic age-associated conditions in humans.

## GRANTS

This study was supported by National Institutes of Health Grants AG047784 (to R. Giosca-Ryan); AG-000279 (to R. Giosca-Ryan); HL-107120-04 (to D. R. Seals) and AG-0138038 (to D. R. Seals). Work in M. P. Murphy’s laboratory is supported by the Medical Research Council UK (MC_U105663142) and by a Wellcome Trust Investigator Award (110159/Z/15/Z).

## DISCLOSURES

M. P. Murphy is on the scientific advisory board of Antipodean Pharmaceuticals. All other authors declare that they have no conflicts of interest.

## AUTHOR CONTRIBUTIONS

R.A.G.-R., M.P.M., and D.R.S. conceived and designed research; R.A.G.-R., M.L.B., L.M.C., and J.SE performed experiments; R.A.G.-R., M.L.B., L.M.C., and J.SE analyzed data; R.A.G.-R., M.L.B., L.M.C., J.S.E., M.P.M., and D.R.S. interpreted results of experiments; R.A.G.-R. prepared figures; R.A.G.-R. drafted manuscript; R.A.G.-R., M.L.B., L.M.C., J.S.E., M.P.M., and D.R.S. edited and revised manuscript; R.A.G.-R., M.L.B., L.M.C., J.S.E., M.P.M., and D.R.S. approved final version of manuscript.
